# Sex Differences in Cognitive Decline Among US Adults

**DOI:** 10.1001/jamanetworkopen.2021.0169

**Published:** 2021-02-25

**Authors:** Deborah A. Levine, Alden L. Gross, Emily M. Briceño, Nicholas Tilton, Bruno J. Giordani, Jeremy B. Sussman, Rodney A. Hayward, James F. Burke, Stephanie Hingtgen, Mitchell S. V. Elkind, Jennifer J. Manly, Rebecca F. Gottesman, Darrell J. Gaskin, Stephen Sidney, Ralph L. Sacco, Sarah E. Tom, Clinton B. Wright, Kristine Yaffe, Andrzej T. Galecki

**Affiliations:** 1 Cognitive Health Services Research Program, Department of Internal Medicine, University of Michigan, Ann Arbor; 2 Department of Neurology and Stroke Program, University of Michigan, Ann Arbor; 3 Institute for Healthcare Policy and Innovation, University of Michigan, Ann Arbor; 4 Department of Epidemiology, Johns Hopkins Bloomberg School of Public Health, Baltimore, Maryland; 5 Department of Physical Medicine and Rehabilitation, University of Michigan, Ann Arbor; 6 Department of Psychiatry, University of Michigan, Ann Arbor; 7 Michigan Alzheimer’s Disease Center, University of Michigan, Ann Arbor; 8 VA Ann Arbor Healthcare System, Ann Arbor, Michigan; 9 Vagelos College of Physicians and Surgeons, Department of Neurology, Columbia University, New York, New York; 10 Mailman School of Public Health, Department of Epidemiology, Columbia University, New York, New York; 11 Taub Institute for Research on Alzheimer’s Disease and the Aging Brain, Columbia University, New York, New York; 12 Department of Neurology, Johns Hopkins University, Baltimore, Maryland; 13 Department of Health Policy and Management, Johns Hopkins Bloomberg School of Public Health, Baltimore, Maryland; 14 Kaiser Permanente Northern California Division of Research, Oakland; 15 Department of Neurology, University of Miami, Miami, Florida; 16 Division of Clinical Research, National Institute of Neurological Disorders and Stroke (NINDS), Bethesda, Maryland; 17 Department of Psychiatry, University of California, San Francisco; 18 Department of Neurology, University of California, San Francisco; 19 Department of Epidemiology, University of California, San Francisco; 20 Department of Biostatistics, University of Michigan, Ann Arbor

## Abstract

**Importance:**

Sex differences in dementia risk are unclear, but some studies have found greater risk for women.

**Objective:**

To determine associations between sex and cognitive decline in order to better understand sex differences in dementia risk.

**Design, Setting, and Participants:**

This cohort study used pooled analysis of individual participant data from 5 cohort studies for years 1971 to 2017: Atherosclerosis Risk in Communities Study, Coronary Artery Risk Development in Young Adults Study, Cardiovascular Health Study, Framingham Offspring Study, and Northern Manhattan Study. Linear mixed-effects models were used to estimate changes in each continuous cognitive outcome over time by sex. Data analysis was completed from March 2019 to October 2020.

**Exposure:**

Sex.

**Main Outcomes and Measures:**

The primary outcome was change in global cognition. Secondary outcomes were change in memory and executive function. Outcomes were standardized as *t* scores (mean [SD], 50 [10]); a 1-point difference represents a 0.1-SD difference in cognition.

**Results:**

Among 34 349 participants, 26 088 who self-reported Black or White race, were free of stroke and dementia, and had covariate data at or before the first cognitive assessment were included for analysis. Median (interquartile range) follow-up was 7.9 (5.3-20.5) years. There were 11 775 (44.7%) men (median [interquartile range] age, 58 [51-66] years at first cognitive assessment; 2229 [18.9%] Black) and 14 313 women (median [interquartile range] age, 58 [51-67] years at first cognitive assessment; 3636 [25.4%] Black). Women had significantly higher baseline performance than men in global cognition (2.20 points higher; 95% CI, 2.04 to 2.35 points; *P* < .001), executive function (2.13 points higher; 95% CI, 1.98 to 2.29 points; *P* < .001), and memory (1.89 points higher; 95% CI, 1.72 to 2.06 points; *P* < .001). Compared with men, women had significantly faster declines in global cognition (−0.07 points/y faster; 95% CI, −0.08 to −0.05 points/y; *P* < .001) and executive function (−0.06 points/y faster; 95% CI, −0.07 to −0.05 points/y; *P* < .001). Men and women had similar declines in memory (−0.004 points/y faster; 95% CI, −0.023 to 0.014; *P* = .61).

**Conclusions and Relevance:**

The results of this cohort study suggest that women may have greater cognitive reserve but faster cognitive decline than men, which could contribute to sex differences in late-life dementia.

## Key Points

**Question:**

Does the risk of cognitive decline among US adults vary by sex?

**Findings:**

In this cohort study using pooled data from 26 088 participants, women, compared with men, had higher baseline performance in global cognition, executive function, and memory. Women, compared with men, had significantly faster declines in global cognition and executive function, but not memory.

**Meaning:**

These findings suggest that women may have greater cognitive reserve but faster cognitive decline than men.

## Teaser

This pooled cohort study used data from 5 population-based cohort studies to investigate whether cognitive decline among US adults varies by sex.

## Introduction

Sex differences in dementia risk are unclear. It is known that women have a greater prevalence of Alzheimer disease (AD) than men, at least partly because women live longer.^1,2,3^ Some, but not all, studies suggest that women have higher incidence of AD.^4,5,6^ Sex differences in biological factors (eg, sex hormones), health factors (eg, cardiovascular risk), and social factors (eg, education levels) are hypothesized to contribute to sex differences in dementia risk.^7,8^ However, most studies have focused on the effects of cardiovascular risk and education on sex disparities in late-life dementia.^9,10^ Whether cognitive trajectories differ by sex after accounting for sex differences in cardiovascular risk and education levels is unknown. Using a pooled cohort^11^ of 5 diverse, well-characterized, population-based cohort studies with repeated objective measures of cognition, we conducted a study to assess sex differences in later-life cognitive trajectories. We hypothesized that women have greater cognitive decline than men after adjusting for potential confounders.

## Methods

### Study Design, Participants, and Measurements

The report follows the Strengthening the Reporting of Observational Studies in Epidemiology (STROBE) reporting guideline for cohort studies. This pooled analysis examined individual participant data from 5 well-characterized prospective cohort studies in the US with repeated measures of cognition: Atherosclerosis Risk in Communities Study (ARIC),^12^ Coronary Artery Risk Development in Young Adults Study (CARDIA),^13^ Cardiovascular Health Study (CHS),^14^ Framingham Offspring Study (FOS),^15^ and Northern Manhattan Study (NOMAS)^16^ for years 1971 to 2017 (eAppendix in the Supplement).

Inclusion criteria included no history of dementia or stroke at each cohort’s baseline (because stroke can alter cognitive trajectory)^17^ and no incidence of dementia or stroke before first cognitive assessment. We excluded participants who reported race other than Black or White because so few participants of other races were reported throughout the study cohorts as to preclude examining the association between other race and the dependent variable. We excluded participants reporting Hispanic ethnicity from NOMAS because other cohorts did not collect information on Hispanic ethnicity or had few participants reporting Hispanic ethnicity; therefore it would be difficult to separate the effect of the NOMAS cohort from the effect of Hispanic ethnicity. We required participants to have 1 or more assessments of cognition and 1 or more measurements of blood pressure (BP) at or before the first measurement of cognition because BP is a risk factor for cognitive decline^11,18^ and varies by sex.^18,19^ The University of Michigan institutional review board approved this study. Participating institutions approved the cohort studies, and participants provided written informed consent.

### Cognitive Function Assessments

Trained cohort staff administered in-person cognitive function tests longitudinally; cognitive tests have been validated in Black and White participants^20,21^ and are consistent with the Vascular Cognitive Impairment Harmonization Standards.^22^ In 3 cohorts (ARIC, NOMAS, and CHS), trained staff also administered tests of global cognitive function (but not tests of memory or executive function) by telephone for participants unable to attend some exam visits. Cognitive tests of global cognition can be measured reliably and precisely over the telephone in adults with comparable results.^23^

In order to resolve the challenge of different cognitive tests administered across the cohorts, we cocalibrated available cognitive test items into factors representing global cognition (ie, global cognitive performance), memory (learning and delayed recall/recognition), and executive function (complex and/or speeded cognitive functions) using item response theory methods (eg, a graded response model) that can accommodate both cognitive information in common across cohorts and test items unique to particular cohorts.^23,24^ Cognitive factor score outcomes, estimated using the regression-based method in Mplus,^25,26^ were set to a *t* score metric (mean [SD] score, 50 [10]) at a participant’s first cognitive assessment; a 1-point difference represents a 0.1-SD difference in the distribution of cognition across the 5 cohorts. Higher cognitive scores indicate better performance (eAppendix in the Supplement). The primary outcome was change in global cognition. Secondary outcomes were change in memory and executive function.

### Covariates

We used covariates measured closest to, but not after, the first cognitive assessment. Demographic characteristics considered included sex, age, race, years of school, and cohort study. Participants self-reported sex and race. Vascular risk factors included alcohol use, cigarette smoking, body mass index (calculated as weight in kilograms divided by height in meters squared), waist circumference, physical activity, fasting glucose, low-density lipoprotein cholesterol, history of atrial fibrillation, and systolic BP. We used systolic BP, summarized as the time-dependent cumulative mean of all BPs before each cognitive measurement, because systolic BP tends to have a stronger association with BP-related outcomes than diastolic BP.^18,27,28^ Long-term cumulative mean systolic BP has improved prediction of clinical outcomes compared with single BP measurements^29^ or mean BP over discrete intervals (eg, ≤1 year, 1 to 5 years) before outcome measurement,^28^ and time-dependent cumulative mean systolic BP is associated with cognitive trajectories.^11^ Cohorts measured current hypertension medication use by evidence of medication bottles and self-report (eAppendix in the Supplement).

### Statistical Analysis

Following a prespecified analysis plan, we compared participant characteristics by sex using a Wilcoxon rank sum test or χ^2^ test as appropriate. We used linear mixed-effects models to estimate changes in each continuous cognitive outcome over time by sex. Because the pooled data involved a small number of cohorts (ie, 5 studies), we associated a fixed effect with cohorts when pooling the data. To estimate sex differences in cognitive changes, models included sex and a sex × follow-up time interaction term. The models included covariates listed in Table 1, and 2-way interaction terms involving follow-up time crossed with age at the time of first cognitive assessment, race, time-dependent cumulative mean systolic BP, and hypertension treatment at the time of first cognitive assessment, and subject-specific random effects for intercepts and slopes. Follow-up time was treated as a continuous measure defined as years since first measurement of each cognitive outcome.

**Table 1.** Characteristics of Participants at First Cognitive Assessment by Sex

Variable^a^	Participants, No. (%)		*P* value
	Women (n = 14 313)	Men (n = 11 775)	
Age at first cognitive assessment, median (IQR), y	58 (51-67)	58 (51-66)	.02
Race^b^			
Black	3636 (25.4)	2229 (18.9)	<.001
White	10 677 (74.6)	9546 (81.1)	
Study cohort			
ARIC	7228 (50.5)	5915 (50.2)	<.001
CARDIA	1921 (13.4)	1476 (12.5)	
CHS	2955 (20.6)	2087 (17.7)	
FOS	1571 (11)	1904 (16.2)	
NOMAS	638 (4.5)	393 (3.3)	
Education			
≤8th grade	996 (7.0)	936 (7.9)	<.001
Grades 9-11	1706 (11.9)	1146 (9.7)	
Completed high school	4657 (32.5)	3264 (27.7)	
Some college but no degree	2299 (16.1)	2048 (17.4)	
≥College graduate	4655 (32.5)	4381 (37.2)	
Alcoholic drinks, No./wk			
None	8712 (60.9)	5391 (45.8)	<.001
1-6	3775 (26.4)	3644 (30.9)	
7-13	1053 (7.4)	1421 (12.1)	
≥14	773 (5.4)	1319 (11.2)	
Current cigarette smoking	2629 (18.4)	2370 (20.1)	<.001
Any physical activity	10 936 (76.4)	9468 (80.4)	<.001
BMI, median (IQR)	27.0 (23.8-31.2)	26.8 (24.3-29.8)	<.001
Waist circumference, median (IQR), cm	93.2 (83-104)	97 (89-105)	<.001
History of atrial fibrillation	214 (1.5)	217 (1.8)	.03
Fasting glucose, median (IQR), mg/dL	97 (90.5-105)	99 (92.4-108)	<.001
LDL cholesterol, median (IQR), mg/dL	127 (104.6-152)	125.8 (104-149)	<.001
Antihypertensive medication use	4552 (31.8)	3275 (27.8)	<.001
Follow-up time from first cognitive assessment, median (IQR), y	8 (5.3-20.7)	7 (5.2-20.2)	<.001
Cumulative mean SBP at first cognitive assessment, median (IQR), mm Hg	136 (125-150)	136 (126-148)	.19
*APOE* ε4 alleles^c^			.10
0	9407 (71.8)	7792 (71.7)	
1	3381 (26.0)	2854 (26.3)	
2	319 (2.4)	222 (2.0)	
Cognitive scores at first assessment, median (IQR)			
General cognitive performance	3 (2-5)	3 (2-5)	<.001
Executive function	54.5 (47.8-59.2)	51.4 (45.2-58.0)	<.001
Memory	52.4 (48.9-55.9)	48.9 (45.7-53.6)	<.001

Abbreviations: ARIC, Atherosclerosis Risk in Communities Study; BMI, body mass index (calculated as weight in kilograms divided by height in meters squared); CARDIA, Coronary Artery Risk Development in Young Adults Study; CHS, Cardiovascular Health Study; FOS, Framingham Offspring Study; IQR, interquartile range; LDL, low-density lipoprotein; NOMAS, Northern Manhattan Study; SBP, systolic blood pressure.

SI conversion factor: To convert glucose to millimoles per liter, multiply by 0.0555; to convert LDL cholesterol to millimoles per liter, multiply by 0.0259.

^a^ Unless stated otherwise, univariate statistics for continuous variables are expressed as median and interquartile range represented by 25th to 75th percentile interval.

^b^ Because few participants reported race other than Black or White, we excluded participants with race/ethnicity of other.

^c^ Subgroups of participants from each cohort had information on the number of *APOE* ε4 alleles resulting in 13 107 women and 10 868 men having these data.

For each outcome, all available cognitive observations were used in the primary analysis except observations after the time of first cohort-adjudicated incident stroke during follow-up, because incident stroke alters the cognitive trajectory.^17^ We evaluated model assumptions by inspecting residual plots. There was no evidence of nonlinear effects of covariates and a significant race × sex × time interaction on cognitive trajectories.

We performed a complete case analysis, excluding a small number of participants (693 of 26 781 [2.59%]) from the analytical data set that had missing values in covariates. Statistical significance for all analyses was set as *P* < .05 in 2-sided tests. All analyses were performed using SAS version 9.4 (SAS Institute).

### Sensitivity Analysis

We repeated analyses (1) including participants’ cognitive observations after the time of incident stroke, (2) after adding kidney function (glomerular filtration rate^30^) and history of myocardial infarction because they may be on the causal pathway, (3) adding the number of *APOE* ε4 alleles and an *APOE* ε4 × follow-up time interaction term, and (4) within cohorts to assess heterogeneity in the associations between sex and cognitive decline.

## Results

**Figure 1.**
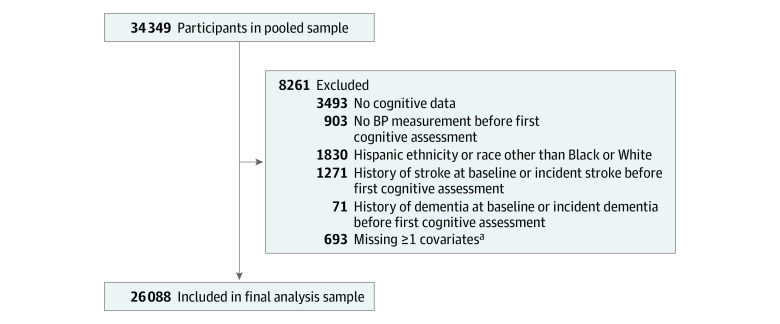
Derivation of Participant Cohort BP indicates blood pressure. ^a^Categories for missing data on covariates are not mutually exclusive. Missing data for covariates included glucose (261 participants), alcohol use (18 participants), body mass index (33 participants), waist circumference (109 participants), smoking (3 participants), physical activity (30 participants), low-density lipoprotein cholesterol (264 participants), antihypertensive medication use (25 participants), and education (179 participants). No participants were missing history of atrial fibrillation.

The study sample included 26 088 participants, 11 775 men (45.1%) (median [interquartile range {IQR}] age, 58 [51-66] years at first cognitive assessment; 2229 [18.9%] Black) and 14 313 women (54.9%) (median [IQR] age, 58 [51-67] years at first cognitive assessment; 3636 [25.4%] Black). Figure 1 shows the derivation of the cohort from the pooled sample. Table 1 presents demographic and clinical characteristics of participants by sex. Most participants completed 2 or more cognitive assessments (22 364 participants [85.7%]). During a median (IQR) follow-up of 7.9 (5.3-20.5) years, the median (IQR) number of global cognition assessments was 3 (2-5) for women and men, the median (IQR) number of executive function assessments was 2 (2-4) for women and men, and the median (IQR) number of memory assessments was 2 (1-3) for women and 2 (2-3) for men. eTable 1 in the Supplement shows characteristics of study participants by cohort. Because the secondary outcome measures were performed less frequently, the executive function analysis included 24 392 participants and the memory analysis included 20 191 participants. eTable 2 in the Supplement has information on missing data and attrition.

### Change in Global Cognition

**Figure 2.**
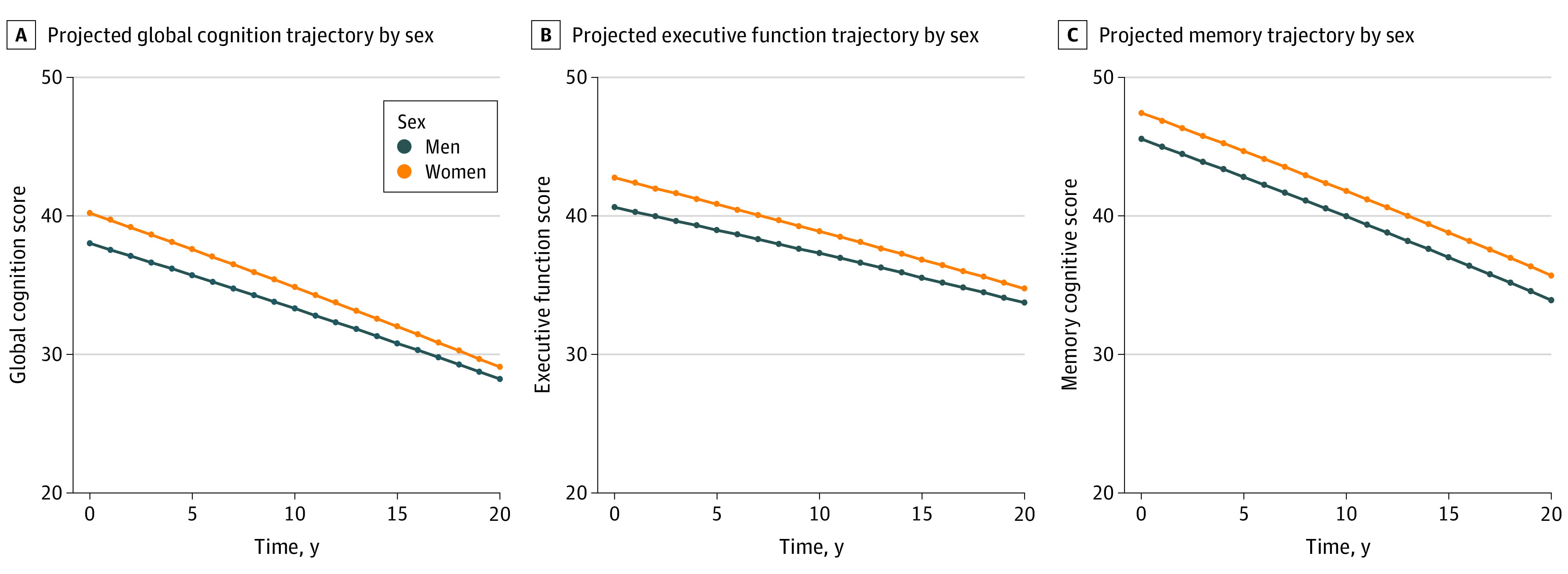
Projected Mean Changes in Global Cognition, Executive Function, and Memory Over Time by Sex Participant-specific (conditional) projected values of cognition were calculated for a 70-year-old Black participant (woman vs man) with the following values of all covariates at or before first cognitive assessment: Northern Manhattan Study cohort, eighth grade or lower education, 0 alcoholic drinks per week, nonsmoking, body mass index of 27.1 (calculated as weight in kilograms divided by height in meters squared), waist circumference (96.0 cm), low-density lipoprotein cholesterol (123.8 mg/dL [to convert to millimoles per liter, multiply by 0.0259]) and glucose (97.3 mg/dL [to convert to millimoles per liter, multiply by 0.0555]), no history of atrial fibrillation, no hypertension treatment, and a baseline systolic blood pressure (BP) of 150 mm Hg that increases by 1 mm each year. Random effects for this projection were set to zero. Linear mixed-effects models included time since first cognitive assessment and baseline values (measured before or at time of first cognitive assessment) of sex, age, race, cohort study, years of school, alcohol use, cigarette smoking, body mass index, waist circumference, physical activity, cumulative mean systolic BP, hypertension treatment, fasting glucose, low-density lipoprotein cholesterol, history of atrial fibrillation, age × follow-up time, sex × follow-up time, race × follow-up time, cumulative mean systolic BP × follow-up time, and hypertension treatment × follow-up time.

Women had significantly higher baseline performance than men in global cognition (2.20 points higher; 95% CI, 2.04 to 2.35 points; *P* < .001) (Table 2). Women, compared with men, had significantly faster declines in global cognition (Figure 2 and Table 2). White men at a median age of 58 years experienced mean declines in global cognition of 0.21 points per year (95% CI, −0.22 to −0.20 point/y; *P* < .001). White women of similar age experienced mean declines in global cognition of 0.27 points per year (95% CI, −0.29 to −0.26 points/y). The adjusted difference in slope was −0.07 points per year faster in women (95% CI, −0.08 to −0.05 points/y; *P* < .001).

**Table 2.** Association of Cognition Decline With Sex Adjusted for Patient Factors^a^

Coefficient	Dependent variables					
	Global cognition (n = 26 088)		Executive function (n = 24 392)		Memory (n = 20 191)	
	Estimate^b^ (95% CI)	*P* value	Estimate^b^ (95% CI)	*P* value	Estimate^b^ (95% CI)	*P* value
At first cognitive assessment						
Difference in intercept between women and men	2.20 (2.04 to 2.35)	<.001	2.13 (1.98 to 2.29)	<.001	1.89 (1.72 to 2.06)	<.001
Change in intercept per 10-y increase in age	−2.09 (−2.20 to −1.98)	<.001	−2.40 (−2.52 to −2.28)	<.001	−1.73 (−1.86 to −1.59)	<.001
Slope in White men at median age, per y	−0.21 (−0.22 to −0.20)	<.001	−0.33 (−0.34 to −0.32)	<.001	−0.23 (−0.25 to −0.21)	<.001
Difference in slope between White women and White men, per y	−0.07 (−0.08 to −0.05)	<.001	−0.06 (−0.07 to −0.05)	<.001	−0.004 (−0.023 to 0.014)	.61
Change in slope per 10-y increase in age at first cognitive assessment, per y	−0.12 (−0.13 to −0.11)	<.001	−0.028 (−0.034 to 0.022)	<.001	−0.16 (−0.18 to −0.15)	<.001

^a^ Linear mixed-effects models included time since first cognitive assessment and baseline values (measured before or at time of first cognitive assessment) of sex, race, age, cohort study, years of school, alcohol use, cigarette smoking, body mass index, waist circumference, physical activity, time-varying cumulative mean systolic blood pressure (BP), hypertension treatment, fasting glucose, low-density lipoprotein (LDL) cholesterol, history of atrial fibrillation, age × follow-up time, sex × follow-up time, race × follow-up time, time-varying cumulative mean systolic BP × follow-up time, and hypertension treatment × follow-up time. To take into account correlation between longitudinal cognitive measures, we included random intercept and slope effects associated with participants. All continuous covariates were centered at the overall median, except cumulative mean systolic BP, which was centered at 120 mm Hg. Glucose, LDL cholesterol, and systolic BP values were divided by 10 so that the parameter estimates refer to a 10-unit change in the variables. Systolic BP was the time-dependent mean of all systolic BPs before the measurement of cognition. To estimate sex differences in cognitive decline, models included a sex × follow-up time interaction term.

^b^ Global cognition measures global cognitive performance. All cognitive measures are set to a *t* score metric (mean 50, SD 10) at a participant’s first cognitive assessment; a 1-point difference represents a 0.1-SD difference in the distribution of cognition across the 5 cohorts. Higher cognitive scores indicate better performance.

### Changes in Executive Function and Memory

Women had significantly higher baseline performance than men in executive function (2.13 points higher; 95% CI, 1.98 to 2.29 points; *P* < .001) and memory (1.89 points higher; 95% CI, 1.72 to 2.06 points; *P* < .001) (Table 2). Compared with men, women had significantly faster declines in executive function (−0.06 points/y faster; 95% CI, −0.07 to −0.05 points; *P* < .001) but not in memory (−0.004 points/y faster; 95% CI, −0.023 to 0.014 points; *P* = .61) (Figure 2 and Table 2).

### Sensitivity Analysis

Results were similar in analyses including participants’ cognitive observations after the time of incident stroke, adding glomerular filtration rate and history of myocardial infarction as covariates, and adding *APOE* ε4 and *APOE* ε4 × time variables as covariates (eTables 3-5 in the Supplement). There was little heterogeneity in the associations between sex and cognitive decline across cohorts (eTable 6 in the Supplement).

## Discussion

Among 26 088 individuals pooled from 5 prospective cohort studies, women had higher baseline performance than men in global cognition, executive function, and memory. Women, compared with men, had significantly faster declines in global cognition and executive function but not memory. These sex differences persisted after accounting for the influence of age, race, education, and cumulative mean BP.

Our results provide evidence suggesting that women have greater cognitive reserve but faster cognitive decline than men, independent of sex differences in cardiovascular risk factors and educational years. Previous studies^31^ have shown that women have higher initial scores on most types of cognitive tests except those measuring visuospatial ability. Few studies have examined sex differences in cognitive trajectories in population-based cohorts of cognitively normal Black and White individuals. A 2016 study^31^ of older adults in Baltimore (mean ages 64-70 years) found that men had steeper rates of decline on 4 of 12 cognitive tests (mental status [Mini Mental State Examination], perceptuomotor speed and integration, visual memory, and visuospatial ability) but no sex differences in declines on 8 of 12 cognitive tests (verbal learning and memory, object recognition and semantic retrieval, fluent language production, attention, working memory and set-shifting, perceptuomotor speed, and executive function). Similarly, we found no sex differences in verbal learning and memory; but, in contrast, we found that women had faster cognitive decline in global cognitive performance and executive function than men. These latter results might differ because we included young and middle-aged adults (mean age 58 years). Our findings are consistent with studies showing that women with mild cognitive impairment or AD have faster decline in global cognition than men.^32,33^

Our results of sex differences in cognitive decline were consistent across most cohorts. The potential reasons for the finding of slower cognitive decline in women in the Framingham Offspring Study are unclear and might be due to socioeconomic, life stress, geographic, and environmental factors as well as cohort differences in sampling strategies, eligibility criteria, and cognitive tests. Although our finding that declines in memory do not differ by sex are consistent with other studies,^31^ the finding is surprising because memory decline is the clinical hallmark of AD, a common cause of dementia,^1^ and some studies suggest that women have higher incidence of AD.^4,5,6^ One explanation is that women manifest verbal memory declines at more advanced stages of neurodegenerative disease than men owing to women having greater initial verbal memory scores and cognitive reserve.^34,35^ However, evidence against this explanation is that women in our study had faster declines in global cognition and executive function despite having higher initial levels of these measures. Another explanation is that the memory measure was less sensitive than the global cognition and executive function measures to detect sex differences in cognitive decline.

If the observed sex differences in declines in global cognition and executive function are causal, then they would be clinically significant, equivalent to 5 to 6 years of cognitive aging. The faster declines in mean cognitive scores associated with female sex can be related to approximate equivalent changes in years of brain or cognitive aging by calculating the ratio of slope coefficients for female sex and baseline age on cognition. Experts have defined clinically meaningful cognitive decline as a decline in cognitive function of 0.5 or more SDs from baseline cognitive scores.^36,37,38^ Women will reach the threshold of a 0.5-SD decrease from the baseline score 4.72 years faster than men for global cognition, 1.97 years faster for executive function, and 0.24 years faster for memory (eTable 7 in the Supplement). Based on this approach, sex differences in cognitive declines are clinically meaningful. Declines in global cognition and executive function markedly raise the risk of death, dementia, and functional disability.^39,40,41^ Diagnosis of the clinical syndrome of dementia/neurocognitive disorder requires cognitive decline by history and objective measurement.^42^ Our findings that women have faster declines in global cognition and executive function mean women would have greater risk than men for being diagnosed with dementia based on objectively measured cognitive decline. Our findings that women had higher initial cognitive scores suggest informants and clinicians might not observe significant cognitive decline in women until substantial loss and impairment has occurred.

Studies have consistently found evidence of sex differences in baseline cognitive functioning with women demonstrating stronger verbal cognitive skills than men, but men demonstrating stronger visuospatial skills than women (eg, mental rotations).^31,43^ Reasons for these sex differences are complex and likely influenced by biological (eg, sex hormones), genetic (eg, *APOE*), and social and cultural factors.^43^ While sex differences in cognitive reserve might also be associated with differences in life course risk factors such as vascular risk,^44^ education, and health behaviors such as smoking and exercise,^45^ our findings of sex differences in baseline cognitive performance independent of these factors suggest that additional contributors and biological pathways play a role.

Women might have faster cognitive decline than men because of differences in sex hormones, structural brain development, genetics, psychosocial factors, lifestyle factors, functional connectivity, and tau pathology.^45,46,47^ Women might have greater burden of small vessel disease, including white matter hyperintensity volume, and less axonal structural integrity that in turn leads to faster cognitive decline particularly in executive function and processing speed.^48,49^ Women also appear to have lower gray matter volume,^50^ so they might be more vulnerable to both the accelerated gray volume loss that occurs with aging and the differential volume loss in specific brain regions that occurs with neurodegenerative diseases.^51^ Recent studies suggest that women develop greater neurofibrillary degeneration, brain parenchymal loss, and cognitive decline.^52,53,54^ Our results suggest that women’s greater cognitive reserve might enable them to withstand greater AD-pathology than men.

### Strengths and Limitations

Our study has several strengths. By pooling 5 large, high-quality cohorts, we had longitudinal cognitive assessments and vascular risk factor measurements in a large number of Black and White individuals who were young, middle-aged, and older-aged to estimate cognitive trajectories in men and women. We had repeated cognitive measures during up to 21 years of follow-up. The cohort studies included in our study systematically measured major cognitive domains important for daily, occupational, and social functioning: global cognition, executive function, and memory. Our findings were consistent across cohorts.

This study also has several limitations. While we adjusted for educational years, we could not adjust for educational quality, literacy, other socioeconomic factors,^10^ or depressive symptoms, because not all cohorts had these data at or before the first cognitive assessment. However, studies suggest that socioeconomic factors tend to influence initial cognitive scores (ie, intercepts) rather than the change in cognitive scores over time (slopes).^55,56^ Selective attrition of cognitively impaired participants could underestimate the rate of cognitive decline^57^ or not.^58^ Estimating the potential clinical impact of sex differences in cognitive decline by correlating it with decline due to aging is a common approach, but it does not directly measure clinical impact, and a clinically meaningful change might vary by an individual’s age, educational quality, race, and baseline cognition.^59^ There were no sex differences in participants excluded because of stroke or dementia before first cognitive assessment, so this would not influence sex differences in cognitive decline (eTable 8 in the Supplement).

We did not study incident dementia because some cohort studies lacked this information. By design, we did not adjust for baseline cognition. We also did not study any particular age interval associated with greatest risk of sex-related cognitive decline. Heterogeneity of the association of sex with cognitive decline between cohorts might have affected the statistical validity of the summary estimate of the effect in the pooled cohort. Smaller sample size and fewer cognitive assessments might have reduced precision of estimates of cognitive decline in executive function and memory (ie, the secondary outcomes). We did not have information on participants’ instrumental activities of daily living, family history of dementia, and hormone replacement therapy use. While the assumption that participants’ postmortem cognitive data are missing at random might lead to immortal cohort bias and underestimate memory declines,^60^ it is valid to answer the research question quantifying sex differences in cognitive trajectories through study follow-up. Women might have had a greater likelihood of regressing to a lower value than men at follow-up because they had higher baseline cognitive function than men. Using a fixed effect for cohorts might have produced conservative estimates of sex effects on cognitive slopes.

## Conclusions

These results suggest that women have greater cognitive reserve but faster later-life cognitive decline than men. Evidence suggests that dementia incidence in Europe and the US has declined over the past 25 years, but declines were less in women than in men.^61^ Our findings suggest that women are at risk for delayed identification of cognitive decline, yet more rapid trajectory of decline, suggesting increased risk of dementia and disability compared with men, consistent with research showing that women with mild cognitive impairment or AD have faster cognitive decline than men.^32,33^ Women may thus have greater needs for caregiving and functional support resources, particularly given women’s longer life expectancy compared with men. Women may also have greater need for serial cognitive assessment to allow for earlier detection of cognitive decline.

## Notes

Supplement. **eAppendix.** Description of Cohort Studies, Harmonization of Cognitive Function Assessments, Cognitive Tests by Domain, Covariates **eTable 1.** Characteristics of Participants at First Cognitive Assessment in Pooled Cohort Sample by Cohort **eTable 2.** Disposition of Patients in the Study **eTable 3.** Sensitivity Analysis of Association of Cognitive Decline With Sex Including Cognitive Observations After Incident Stroke **eTable 4.** Sensitivity Analysis of Association of Cognitive Decline With Sex Including Kidney Function and History of Myocardial Infarction as Covariates **eTable 5.** Sensitivity Analysis of Association of Cognitive Decline with Sex Including Number of APOE 4 Alleles as a Covariate **eTable 6.** Association of Cognitive Decline With Sex Over Time by Cohort **eTable 7.** Time to Reach the Threshold of 0.5 Standard Deviation Decrease in Cognitive Function From Baseline by Sex **eTable 8.** Association Between Sex and Exclusion Because of History of Stroke or Dementia at Baseline or Incident Stroke or Incident Dementia Before First Cognitive Assessment **eReferences.**
