# Error in Methods

**DOI:** 10.1001/jamanetworkopen.2023.4786

**Published:** 2023-03-06

In the Original Investigation titled “Sex Differences in Cognitive Decline Among US Adults,”^1^ published February 25, 2021, there was an error in the description of the analytic sample in the Methods. Eligible patients were required to have 1 or more measurements of blood pressure before the measurement of cognition. This article has been corrected.^1^
