# Supplementary material for: Sex Differences in Cognitive Decline Among US Adults
Source: JAMA Netw Open. 2021 Feb 25;4(2):e210169. doi: 10.1001/jamanetworkopen.2021.0169 (PMC7907956; doi:10.1001/jamanetworkopen.2021.0169)
Supplement: Supplement. — eAppendix. Description of Cohort Studies, Harmonization of Cognitive Function Assessments, Cognitive Tests by Domain, Covariates eTable 1. Characteristics of Participants at First Cognitive Assessment in Pooled Cohort Sample by Cohort eTable 2. Disposition of Patients in the Study eTable 3. Sensitivity Analysis of Association of Cognitive Decline With Sex Including Cognitive Observations After Incident Stroke eTable 4. Sensitivity Analysis of Association of Cognitive Decline With Sex Including Kidney Function and History of Myocardial Infarction as Covariates eTable 5. Sensitivity Analysis of Association of Cognitive Decline with Sex Including Number of APOE 4 Alleles as a Covariate eTable 6. Association of Cognitive Decline With Sex Over Time by Cohort eTable 7. Time to Reach the Threshold of 0.5 Standard Deviation Decrease in Cognitive Function From Baseline by Sex eTable 8. Association Between Sex and Exclusion Because of History of Stroke or Dementia at Baseline or Incident Stroke or Incident Dementia Before First Cognitive Assessment eReferences. [file jamanetwopen-e210169-s001.pdf]

Supplemental Online Content

Levine DA, Gross AL, Briceño EM, et al. Sex differences in cognitive decline among US adults. *JAMA Netw Open*. 2021;4(2):e210169. doi:10.1001/jamanetworkopen.2021.0169

eAppendix. Description of Cohort Studies, Harmonization of Cognitive Function Assessments, Cognitive Tests by Domain, Covariates

eTable 1. Characteristics of Participants at First Cognitive Assessment in Pooled Cohort Sample by Cohort

eTable 2. Disposition of Patients in the Study

eTable 3. Sensitivity Analysis of Association of Cognitive Decline With Sex Including Cognitive Observations After Incident Stroke

eTable 4. Sensitivity Analysis of Association of Cognitive Decline With Sex Including Kidney Function and History of Myocardial Infarction as Covariates

eTable 5. Sensitivity Analysis of Association of Cognitive Decline with Sex Including Number of APOE4 Alleles as a Covariate

eTable 6. Association of Cognitive Decline With Sex Over Time by Cohort

eTable 7. Time to Reach the Threshold of 0.5 Standard Deviation Decrease in Cognitive Function From Baseline by Sex

eTable 8. Association Between Sex and Exclusion Because of History of Stroke or Dementia at Baseline or Incident Stroke or Incident Dementia Before First Cognitive Assessment

eReferences.

This supplemental material has been provided by the authors to give readers additional information about their work.

eMethods

Cohort Studies

The Coronary Artery Risk Development in Young Adults study (CARDIA) (http://www.cardia.dopm.uab.edu/) is a study on development of and risk factors for cardiovascular disease (CVD) in young adults. In 1985-1986, the study recruited 5,115 adults aged 18-30 (50% black, 50% white) from 4 US cities (Birmingham, AL; Chicago, IL; Minneapolis, MN; Oakland, CA). Within sites, the sample aimed to comprise ~equal numbers of participants by sex, self-defined race (black or white), age (18 to 24 or 25 to 30 years), and education (≤high school or >high school). Follow-up exams have occurred every 2-5 years for 30 years. Exams at years 25 and 30 measured cognition.

The Northern Manhattan Study (NOMAS) (http://columbianomas.org/index.html) is a study on determinants of stroke and CVDs in middle-aged and older adults in 3 race-ethnic groups. From 1993-2001, the study recruited 3,298 stroke-free adults aged 40 or older (20% black, 25% white, 55% Hispanic) from Northern Manhattan. NOMAS has a longitudinal dementia sub-study (n=1,290) that expanded the neuropsychology battery; these participants have had follow-up exams every 5 years for 15 years (3rd exam approximately 2016-2020) and cognition measured at each exam. We included the expanded neuropsychology battery and the dementia sub-study in the analysis.

The Atherosclerosis Risk in Communities Study (ARIC) (https://www2.cscc.unc.edu/aric/desc) is a study of the causes of atherosclerosis and its clinical outcomes, and variation in CVD risk factors, medical care, and disease by race, gender, location, and date. In 1987-1989, the study recruited 15,792 adults aged 45-64 (28% black, 72% white) from 4 US communities (Forsyth County, NC, Jackson, MS,

© 2021 Levine DA et al. *JAMA Network Open.*

Minneapolis, MN, Washington County, MD). Participants have had follow-up exams every 2-13 years for 25+ years. Starting at the 2nd exam, every exam measured cognition (n=14,348) using a short battery. We did not use data from the 3rd exam, when only a very small proportion of the cohort had cognition assessed. ARIC has a neurocognitive sub-study to perform CID surveillance. ARIC expanded the neuropsychology battery in exams 5-7. We included the neurocognitive sub-study and expanded neuropsychology battery in the analysis.

The Cardiovascular Health Study (CHS) (http://www.chs-nhlbi.org/) is a study of risk factors for CVD in older adults. In 1989-1990, the study recruited 5,201 adults aged ≥65 (16% black, 84% white), and in 1992-1993, added a supplementary cohort of 687 blacks, from 4 US communities (Sacramento County, CA, Washington County, MD, Forsyth County, NC, Pittsburgh, PA). We used in-person cognitive test data from study years 3-11 and TICS data from years 8-11 and 20-25. Data on test timing (days from baseline) were not available after year 25. CHS also had a dementia sub-study that added a neuropsychology battery and neurology exam, which we included in the analysis.

The Framingham Offspring Study (FOS) (https://www.framinghamheartstudy.org/index.php) is a study of risk factors for CVD. In 1971, the study recruited 5,124 adults aged <10-70 (>95% white) from New England. Follow-up exams have occurred every 4-8 years for 45 years. All exams measured cognition using: MMSE at all exams and an expanded neuropsychology battery at exam 7 forward. In 1999-2000, FOS began a cognitive and MRI sub-study that added an expanded neuropsychology battery. We removed 9 non-Hispanic blacks to avoid

© 2021 Levine DA et al. *JAMA Network Open.*

working with a qualitative variable containing a category with a small number of subjects. We included the expanded neuropsychology battery and the cognitive and MRI sub-study in the analysis. We included FOS because it contributes individual participant data to estimate cognitive decline in whites—the reference group in the pooled cohort analysis to which blacks are compared—as well as the effect of time-dependent cumulative mean BP on cognitive decline.

Harmonization of Cognitive Function Assessments

In a pre-statistical harmonization phase, we identified 126 test items from 32 cognitive instruments across the cohorts and determined shared items between cohorts.[1] Expert neuropsychologists (EMB, BJG) assigned each test item to a cognitive domain. This phase entailed extensive review of study-specific scoring procedures, test administration, and documentation of theoretical and empirical score ranges of each cognitive test score in each study. The goal was to identify comparable and non-comparable tests across studies. Neuropsychologists paid attention to versions of tests (e.g., WAIS vs WMS). We used equipercentile equating to adjust the distributions of like test items across cohorts (e.g., Auditory Verbal Learning Test and the California Verbal Learning Test) to be on a common scale prior to IRT co-calibration.[2] In equipercentile equating, each percentile rank of a score on one study is equated to the score with the same percentile in another study; the determination of which tests were truly comparable in quality was made by the expert clinicians. Decisions regarding how specific cognitive test variables were treated are available from the corresponding author.

© 2021 Levine DA et al. *JAMA Network Open.*

For statistical harmonization we used item response theory (IRT). IRT describes a family of statistical methods that are used to relate discrete observations (e.g., cognitive tests) to a latent trait that underlies the observations[3]. In an IRT model, each test item is empirically weighted based on its correlation with other items and assigned a relative location along the latent trait (e.g., global cognition) corresponding to its estimated difficulty. This weighting and calculation of relative location is encapsulated in item discrimination (or loading) and local (or threshold) parameters, which are estimated simultaneously alongside a person's ability level and expressed on a unified metric via maximum likelihood during the model estimation process. We computed factor scores from IRT-graded response models for each domain using the regression-based method in Mplus version 8.1.[4-7] Using the regression method - also known as the empirical Bayes predictor[8] - the factor score is computed on the basis of all available information in a given observation's response patterns and thus can be calculated in the presence of missing data. This IRT approach produces cognitive scores that are more precise than those derived from a z-score approach that standardizes and averages test scores.[9] Details are available in a forthcoming publication. The measurement model for each cognitive factor was constructed such that cognitive tests provided no information about cognitive levels at study visits in which the tests were not administered. Although the cognitive factors were constructed in this way, there remains an autocorrelation across time within people that is modeled using random effects models described in the main analysis section.

© 2021 Levine DA et al. *JAMA Network Open.*

Cognitive Tests by Domain

Global cognition domain: Animal Naming (ARIC, CARDIA, CHS, FOS, NOMAS), Baddeley and Papagno Divided Attention Task (CHS), Block Design Test (FOS: WAIS; CHS: WAIS-R), Boston Naming Test (ARIC, CHS, FOS, NOMAS), Color Trails Test (NOMAS), modified California Verbal Learning Test (CHS, NOMAS), Delayed Word Recall test (ARIC), Developmental Test of Visuomotor Integration (NOMAS), Digit Ordering Test (NOMAS), Digit Span Test (ARIC: WMS-R; CHS: WAIS-R; FOS: WAIS), Digit Symbol Substitution Test/Digit Symbol Coding Test (CARDIA: WAIS-III; ARIC, CHS: WAIS-R), Finger Tapping Test (FOS, ARIC), Grooved Pegboard Test (CHS, NOMAS), Hooper Visual Organization Test (FOS), Letter Fluency Test (ARIC, CARDIA, CHS, FOS, NOMAS), Letter-Number Sequencing (WAIS-III; NOMAS), Logical Memory Test (ARIC: WMS-R; FOS: WMS), Mini Mental State Examination (ARIC, CHS, FOS, NOMAS), Modified Mini Mental State Examination (CHS), Montreal Cognitive Assessment (CARDIA), Odd Man Out (NOMAS), Paired Associates Learning (WMS; FOS), Ravens Colored Progressive Matrices (CHS), Rey Auditory Verbal Learning Test (CARDIA), Rey-Osterrieth Complex Figure Test (CHS), Semantic Word Generation (CHS), Similarities (WAIS; FOS), Stroop Neuropsychological Screening Test (CHS)/Stroop Test (CARDIA), Symbol Digit Modalities Test (NOMAS), Telephone Interview for Cognitive Status (ARIC, CHS, NOMAS), Trail Making Test (ARIC, CHS, FOS), Visual Reproduction (WMS; FOS).

Memory domain: modified California Verbal Learning Test (NOMAS), Delayed Word Recall test (ARIC), Digit Symbol Substitution Test (Incidental Learning; ARIC); Logical

© 2021 Levine DA et al. *JAMA Network Open.*

Memory Test (ARIC: WMS-R; FOS: WMS), Paired Associates Learning (WMS; FOS), Rey Auditory Verbal Learning Test (CARDIA), Visual Reproduction (WMS; FOS).

Executive function domain: Animal Naming (ARIC, CARDIA, CHS, FOS, NOMAS), Baddeley and Papagno Divided Attention Task (CHS), Color Trails Test (NOMAS), modified California Verbal Learning Test (CHS; semantic clustering), Digit Ordering Test (NOMAS), Digit Span Test (ARIC: WMS-R; CHS: WAIS-R; FOS: WAIS), Digit Symbol Substitution Test/Digit Symbol Coding Test (CARDIA: WAIS-III; ARIC, CHS: WAIS-R), Letter Fluency Test (ARIC, CARDIA, CHS, FOS, NOMAS), Letter-Number Sequencing (WAIS-III; NOMAS), Odd Man Out (NOMAS), Semantic Word Generation (CHS), Similarities (WAIS; FOS), Stroop Neuropsychological Screening Test (CHS)/Stroop Test (CARDIA), Symbol Digit Modalities Test (NOMAS), Trail Making Test (ARIC, CHS, FOS)

Note: WAIS is Wechsler Adult Intelligence Scale. WAIS-R is Wechsler Adult Intelligence Scale-Revised. WAIS-III is Wechsler Adult Intelligence Scale-3rd Edition. WMS is Wechsler Memory Scale. WMS-R is Wechsler Memory Scale -Revised. WMS-III is Wechsler Memory Scale -3rd Edition.

Cognitive tests were administered based on the protocols and schedules of the cohort studies and their ancillary studies. Information on the timing of the administration of each cognitive test by cohort is available on request.

© 2021 Levine DA et al. *JAMA Network Open.*

Covariates

We harmonized covariates across cohorts by choosing common response categories for categorical variables and converting measurements to common units for continuous variables. The cohorts used experts to adjudicate incident clinical events during follow-up (e.g., stroke, myocardial infarction, atrial fibrillation). Years of school was categorized as eighth grade or less, grades 9-11, completed high school, some college but no degree, and college graduate or more, and cohort study. Alcohol use was categorized as drinks per week (none, 1-6, 7-13, 14 or more). Cigarette smoking was defined as current or not. Waist circumference was measured in centimeters. Assessment of physical activity varied across the cohorts. CHS and NOMAS asked participants about activity over the previous two weeks. CARDIA and ARIC created summary scores from questions about frequency of specific activities over the previous year. FOS asked participants to provide the number of hours per day of moderate or heavy exercise. A harmonized binary variable was created by assigning a “yes” value to any participants indicating that they performed any activity over the respective time periods. Low-density lipoprotein (LDL) cholesterol and fasting glucose were measured in mg/dL. Fasting glucose levels were provided by ARIC, CARDIA and FOS. For CHS and NOMAS, glucose levels were provided along with separate fasting indicators. In CHS data, the number of hours since eating was provided, and we defined fasting as having gone 8 or more hours since eating. In NOMAS data, the fasting indicator was a binary variable defined as fasting vs. non-fasting, so we could not consider the number of hours since eating. In both CHS and NOMAS, glucose levels were treated as missing if not fasting. History of atrial fibrillation at or before the first cognitive assessment was defined as

© 2021 Levine DA et al. *JAMA Network Open.*

history of atrial fibrillation based on self-report and/or physician adjudication at baseline or cohort-adjudicated incident atrial fibrillation before the first cognitive assessment. Cohorts measured current hypertension medication use by evidence of medication bottles and self-report. History of myocardial infarction at or before the first cognitive assessment was defined as history of myocardial infarction based on self-report and/or physician adjudication at baseline or cohort-adjudicated incident myocardial infarction before the first cognitive assessment. Kidney function (glomerular filtration rate) was measured using the Chronic Kidney Disease Epidemiology Collaboration (CKD-EPI) equation. History of stroke at or before the first cognitive assessment was defined as history of stroke based on self-report and/or physician adjudication at baseline or cohort-adjudicated incident stroke before the first cognitive assessment. We summarized systolic blood pressure (BP) as the time-dependent cumulative mean of all BPs before each cognitive measurement. It is a “running average” of the systolic BP measurements before each cognitive measurement.

Missing data on the cognitive measures (the dependent variable) measured longitudinally exhibit non-monotone missing data patterns, making the likelihood-based methods challenging to implement if missing data result from the missing not at random (MNAR) mechanism. For this reason, we elected to proceed with the analysis using linear mixed-effects models, which allow for a valid inference assuming that data on cognition are missing at random (MAR).

© 2021 Levine DA et al. *JAMA Network Open.*

eTable 1: Characteristics of Participants at First Cognitive Assessment in Pooled Cohort Sample by Cohort

Variable*	ARIC	CARDIA	CHS	FOS	NOMAS
Study sample, No. (%)	13143 (50.4%)	3397 (13.0%)	5042 (19.3%)	3475 (13.3%)	1031 (4.0%)
Blacks, No. (%)	3044 (23.2%)	1562 (46.0%)	704 (14.0%)	0 (0.0%)	555 (53.8%)
Whites, No. (%)	10099 (76.8%)	1835 (54.0%)	4338 (86.0%)	3475 (100.0%)	476 (46.2%)
Age at first BP measurement, full range, y	44 – 66	17 – 34	64 – 95	5 – 63	40 – 99
Age at first BP measurement, y	54 (49, 59)	25 (22, 28)	71 (68, 76)	34 (27, 42)	71 (64, 77)
Age at first cog assessment full range, y	46 – 88	41 – 61	64 – 96	25 – 83	40 – 103
Age at first cog assessment, y	57 (52, 62)	50 (47, 53)	72 (69, 77)	53 (47, 61)	74 (67, 81)
Women, No. (%)	7228 (55.0%)	1921 (56.6%)	2955 (58.6%)	1571 (45.2%)	638 (61.9%)
Education, No. (%)					
Eighth grade or less	1080 (8.2%)	7 (0.2%)	707 (14.0%)	34 (1.0%)	104 (10.1%)
Grades 9-11	1740 (13.2%)	142 (4.2%)	697 (13.8%)	146 (4.2%)	127 (12.3%)
Completed high school	4363 (33.2%)	650 (19.1%)	1416 (28.1%)	1209 (34.8%)	283 (27.5%)
Some college but no degree	1131 (8.6%)	937 (27.6%)	1166 (23.1%)	898 (25.8%)	215 (20.9%)
College graduate or more	4829 (36.7%)	1661 (48.9%)	1056 (20.9%)	1188 (34.2%)	302 (29.3%)
Alcoholic drinks per week, No. (%)					
None	8631 (65.7%)	1537 (45.3%)	2484 (49.3%)	1064 (30.6%)	387 (37.5%)
One to six	2603 (19.8%)	1058 (31.2%)	1858 (36.9%)	1396 (40.2%)	504 (48.9%)
Seven to thirteen	1139 (8.7%)	461 (13.6%)	303 (6.0%)	510 (14.7%)	61 (5.9%)
Fourteen or more	770 (5.9%)	341 (10.0%)	397 (7.9%)	505 (14.5%)	79 (7.7%)
Current cigarette smoking, No. (%)	2958 (22.5%)	642 (18.9%)	555 (11.0%)	670 (19.3%)	174 (16.9%)
Any physical activity, No. (%)	8488 (64.6%)	3311 (97.5%)	4640 (92.0%)	3255 (93.7%)	710 (68.9%)
Body mass index	27.2 (24.3, 30.7)	28.1 (24.5, 32.9)	26.1 (23.5, 29.1)	26.6 (23.8, 29.8)	26.8 (23.9, 30.3)
Waist circumference, cm	97 (89, 106)	90.8 (80.5, 101.5)	94 (85.5, 102)	92.7 (81.3, 101.6)	91.4 (83.8, 101.6)
History of atrial fibrillation, No. (%)	111 (0.9%)	0 (0.0%)	182 (3.6%)	38 (1.1%)	100 (9.7%)
Fasting glucose, mg/dL	99 (93, 107)	94.5 (88, 101.4)	101 (94, 111)	95 (89, 102)	91 (81, 104)
LDL cholesterol, mg/dL	131.2 (109, 155.4)	109 (88, 130)	128 (106.8, 151.8)	125 (104, 148)	127 (104.4, 150.4)

© 2021 Levine DA et al. *JAMA Network Open.*

Variable*	ARIC	CARDIA	CHS	FOS	NOMAS
Anti-hypertensive medication use, No. (%)	3496 (26.6%)	564 (16.6%)	2627 (52.1%)	565 (16.3%)	575 (55.8%)
Follow-up time from first cognitive assessment, y	19.2 (5.9, 24)	5.2 (4.9, 5.5)	8 (5, 15)	18.5 (13.3, 19.9)	6.1 (1.9, 11.1)
SBP cumulative mean (SD) at first cognitive assessment, mm Hg	139 (129, 150)	127.3 (121.9, 133.1)	148 (136, 163)	119.8 (111.7, 129.9)	140 (130, 155)
Total number of BP measurements	3 (3, 4)	8 (7, 8)	7 (5, 9)	8 (6, 8)	1 (1, 2)
Number of SBP measurements before first cognitive assessment	1 (1, 1)	7 (6, 7)	1 (1, 1)	4 (4, 4)	1 (1, 1)
Time from first SBP measurement to first cognitive assessment, y	2.9 (2.8, 3)	24.7 (24.4, 25.1)	1 (0.9, 1)	19.4 (19.2, 19.6)	3.1 (1.3, 6)
**Cognitive scores at first assessment**					
General cognitive performance	52.7 (46.9, 57.4)	52.7 (46.5, 58.6)	48.5 (43.4, 52.8)	53.5 (52, 58.7)	48.2 (42.6, 54)
Executive function	52.7 (46.3, 57.3)	55.8 (49.9, 61.8)	49.5 (44.4, 55.1)	61.5 (59.3, 64)	54 (47.5, 60.3)
Memory	52.4 (48.9, 55.9)	49.9 (44.2, 56.8)	48.7 (41.8, 57.2)	51.1 (46.3, 55.9)	49.7 (43.1, 56.5)
**Number of cognitive assessments**					
General cognitive performance	2 (2, 3)	2 (2, 2)	9 (5, 10)	6 (4, 7)	5 (2, 8)
Executive function	2 (2, 3)	2 (2, 2)	8 (5, 9)	2 (1, 3)	2 (1, 2)
Memory	2 (2, 3)	2 (2, 2)	1 (1, 1)	2 (1, 3)	2 (1, 2)

Abbreviations: NA=not applicable. SBP=systolic blood pressure. CHS has only one memory measurement.

* Unless stated otherwise, univariate statistics for continuous variables are expressed as median and interquartile range (IQR), where IQR is represented by (lower quartile, upper quartile) interval.

eTable 2: Disposition of patients in the study (Total number of patients N=26,088)

**Follow-up time interval from patients' first cognitive assessment (years)**	**Alive / in study at start of interval**	**Lost to follow-up**		**Died**		**Attrition: died and lost to follow-up**	
	N	N	%	N	%	N	%
[0 , 5)	26,088	3,560	13.6	1,264	4.8	4,824	18.5
[5 , 10)	20,581	4,771	23.2	992	4.8	5,763	28.0
[10 , 15)	11,492	375	3.3	63	0.5	438	3.8
[15 , 20)	10,416	668	6.4	77	0.7	745	7.2
[20 , 25)	7,548	2,226	29.5	577	7.6	2,803	37.1
>= 25	2,476	0	0.0	0	0.0	0	0.0
**Total**		11,600	44.5	2,973	11.4	14,573	55.9

eTable 2 provides information on the patients' attrition. Loss-to follow-up was determined based on cohort-specific cognitive assessment schedules. Overall, 55.9% attrition (those not completing the final cognitive assessments) is high due to the long follow-up time. The number of patients who had known vital status but not complete serial cognitive testing (those missing one or more assessments at any time) was 15,083 (57.8%). Still, most patients had two or more cognitive assessments and contributed information to the sexes' slope estimates. Among the 26,088 patients in the study sample, the number of patients completing two or more cognitive assessments was 22,364 (85.7%). Many patients had long-term follow-up (5+ years). The median (Q1, Q3) follow-up time for patients who were alive through follow-up (n=23,115) was 8.1 (5.5, 20.7) years. Similarly, the median (Q1, Q3) follow-up time for patients who died during cognitive follow-up (n=2,973) was 5.9 (0, 8.0) years. During follow-up, 2,973 patients out of 26,088 (11.4%) have died. We treated the absence of post-mortem cognitive testing data as data missing at random (MAR).

© 2021 Levine DA et al. *JAMA Network Open.*

Table 3: Sensitivity Analysis of Association of Cognitive Decline with Sex Including Cognitive Observations after Incident Stroke

	Global Cognition (n=26,088)		Executive Function (n=24,422)		Memory (n=20,293)	
**Coefficient**	**Estimate (95% CI)**	***P***	**Estimate (95% CI)**	***P***	**Estimate (95% CI)**	***P***
Change in intercept per 10 y increase in age at first cognitive assessment	-2.09 (-2.20, -1.97)	<.001	-2.39 (-2.51, -2.27)	<.001	-1.75 (-1.88, -1.61)	<.001
Difference in intercept between women and men at first cognitive assessment	2.20 (2.05, 2.36)	<.001	2.13 (1.98, 2.29)	<.001	1.89 (1.72, 2.07)	<.001
Change in slope per 10 y increase in age at first cognitive assessment, per y	-0.13 (-0.14, -0.12)	<.001	-0.032 (-0.039, -0.027)	<.001	-0.16 (-0.18, -0.15)	<.001
Slope in white men at median age, per y	-0.22 (-0.23, -0.21)	<.001	-0.33 (-0.34, -0.32)	<.001	-0.23 (-0.25, -0.21)	<.001
Difference in slope between white women and white men, per y	-0.07 (-0.08, -0.05)	<.001	-0.057 (-0.064, -0.045)	<.001	-0.006 (-0.024, 0.012)	0.51

Interpretative Key: Global cognition measures global cognitive performance. All cognitive measures are set to a T-score metric (mean 50, SD 10); a 1-point difference represents a 0.1 SD difference in the distribution of cognition across the 5 cohorts. Higher cognitive scores indicate better performance. SBP= systolic blood pressure. NA=not applicable. y=year.

Linear mixed-effects models included time since first cognitive assessment and baseline values (measured before or at time of first cognitive assessment) of sex, race, age, cohort study, years of school, alcohol use, cigarette smoking, body mass index, waist circumference, physical activity, time-varying cumulative mean systolic blood pressure (SBP), hypertension treatment, fasting glucose, low density lipoprotein (LDL) cholesterol, history of atrial fibrillation, age* follow-up time, sex* follow-up time, race* follow-up time, time-varying cumulative mean SBP* follow-up time, and hypertension treatment*follow-up time.. To take into account correlation between longitudinal cognitive measures, we included random intercept and slope effects associated with subjects. All continuous variables were centered at the overall median, except cumulative mean SBP, which was centered at 120 mmHg. Glucose, LDL cholesterol, and SBP values were divided by 10 so that the parameter estimates refer to a 10-unit change in the variables. SBP was the time-dependent mean of all SBPs before the measurement of cognition. To estimate sex differences in cognitive decline, models included a sex*follow-up time interaction term.

© 2021 Levine DA et al. *JAMA Network Open.*

eTable 4: Sensitivity Analysis of Association of Cognitive Decline with Sex Including Kidney Function and History of Myocardial Infarction as Covariates

	Global Cognition (n=25,973)		Executive Function (n=24,308)		Memory (n=20,107)	
**Coefficient**	**Estimate (95% CI)**	***P***	**Estimate (95% CI)**	***P***	**Estimate (95% CI)**	***P***
Change in intercept per 10 y increase in age at first cognitive assessment	-2.03 (-2.15, -1.91)	<.001	-2.41 (-2.53, -2.29)	<.001	-1.65 (-1.79, -1.51)	<.001
Difference in intercept between women and men at first cognitive assessment	2.26 (2.10, 2.42)	<.001	2.10 (1.95, 2.26)	<.001	1.99 (1.82, 2.17)	<.001
Change in slope per 10 y increase in age at first cognitive assessment, per y	-0.12 (-0.13, -0.11)	<.001	-0.028 (-0.034, -0.022)	<.001	-0.16 (-0.18, -0.15)	<.001
Slope in white men at median age, per y	-0.21 (-0.22, -0.20)	<.001	-0.33 (-0.34, -0.32)	<.001	-0.23 (-0.25, -0.21)	<.001
Difference in slope between white women and white men, per y	-0.065 (-0.078, -0.052)	<.001	-0.056 (-0.065, -0.047)	<.001	-0.006 (-0.024, 0.013)	0.54

Interpretative Key: Global cognition measures global cognitive performance. All cognitive measures are set to a T-score metric (mean 50, SD 10); a 1-point difference represents a 0.1 SD difference in the distribution of cognition across the 5 cohorts. Higher cognitive scores indicate better performance. SBP= systolic blood pressure. NA=not applicable. y=year.

Linear mixed-effects models included time since first cognitive assessment and baseline values (measured before or at time of first cognitive assessment) of sex, race, age, cohort study, years of school, alcohol use, cigarette smoking, body mass index, waist circumference, physical activity, time-varying cumulative mean systolic blood pressure (SBP), hypertension treatment, fasting glucose, low density lipoprotein (LDL) cholesterol, history of atrial fibrillation, age* follow-up time, sex* follow-up time, race* follow-up time, time-varying cumulative mean SBP* follow-up time, and hypertension treatment*follow-up time. To take into account correlation between longitudinal cognitive measures, we included random intercept and slope effects associated with subjects. All continuous variables were centered at the overall median, except cumulative mean SBP, which was centered at 120 mmHg. Glucose, LDL cholesterol, and SBP values were divided by 10 so that the parameter estimates refer to a 10-unit change in the variables. SBP was the time-dependent mean of all SBPs before the measurement of cognition. To estimate sex differences in cognitive decline, models included a sex*follow-up time interaction term.

© 2021 Levine DA et al. *JAMA Network Open.*

eTable 5: Sensitivity Analysis of Association of Cognitive Decline with Sex Including Number of APOE4 Alleles as a Covariate

	Global Cognition (n=23,975)		Executive Function (n=22,686)		Memory (n=18,902)	
**Coefficient**	**Estimate (95% CI)**	***P***	**Estimate (95% CI)**	***P***	**Estimate (95% CI)**	***P***
Change in intercept per 10 y increase in age at first cognitive assessment	-1.99 (-2.11, -1.87)	<.001	-2.38 (-2.51, -2.26)	<.001	-1.71 (-1.85, -1.57)	<.001
Difference in intercept between women and men at first cognitive assessment	2.19 (2.03, 2.35)	<.001	2.12 (1.96, 2.28)	<.001	1.82 (1.65, 2.00)	<.001
Change in intercept per 1 unit increase in no. APOE E4 alleles	-0.22 (-0.37, -0.07)	0.004	-0.24 (-0.39, -0.09)	0.002	-0.19 (-0.36, -0.03)	0.02
Change in slope per 10 y increase in age at first cognitive assessment, per y	-0.12 (-0.13, -0.11)	<.001	-0.029 (-0.036, -0.023)	<.001	-0.16 (-0.18, -0.15)	<.001
Slope in white men at median age, per y	-0.18 (-0.20, -0.17)	<.001	-0.32 (-0.33, -0.31)	<.001	-0.21 (-0.24, -0.19)	<.001
Difference in slope between white women and white men, per y	-0.064 (-0.077, -0.050)	<.001	-0.057 (-0.067, -0.048)	<.001	-0.006 (-0.025, 0.013)	0.53
Change in slope per 1 unit increase in no. APOE E4 alleles	-0.10 (-0.11, -0.08)	<.001	-0.035 (-0.045, -0.026)	<.001	-0.08 (-0.09, -0.06)	<.001

Interpretative Key: Global cognition measures global cognitive performance. All cognitive measures are set to a T-score metric (mean 50, SD 10); a 1-point difference represents a 0.1 SD difference in the distribution of cognition across the 5 cohorts. Higher cognitive scores indicate better performance. SBP= systolic blood pressure. NA=not applicable. No.=number. y=year.

Linear mixed-effects models included time since first cognitive assessment and baseline values (measured before or at time of first cognitive assessment) of sex, race, age, cohort study, years of school, alcohol use, cigarette smoking, body mass index, waist circumference, physical activity, number of APOE E4 alleles, time-varying cumulative mean systolic blood pressure (SBP), hypertension treatment, fasting glucose, low density lipoprotein (LDL) cholesterol, history of atrial fibrillation, age* follow-up time, sex* follow-up time, race* follow-up time, number of APOE E4 alleles*follow-up time, time-varying cumulative mean SBP* follow-up time, and hypertension treatment*follow-up time. To take into account correlation between longitudinal cognitive measures, we included random intercept and slope effects associated with subjects. All continuous variables were centered at the overall median, except cumulative mean SBP, which was centered at 120 mmHg. Glucose, LDL cholesterol, and SBP values were divided by 10 so that the parameter estimates refer to a 10-unit change in the variables. SBP was the time-dependent mean of all SBPs before the measurement of cognition. To estimate sex differences in cognitive decline, models included a sex*follow-up time interaction term.

© 2021 Levine DA et al. *JAMA Network Open.*

eTable 6: Association of Global Cognitive Decline with Sex over Time by Cohort

Variables	ARIC (n=13,143)	CHS (n=5,042)	FOS (n=3,475)	NOMAS (n=1,031)
Slope in white men at median age, per y	-0.295 (-0.312, -0.277)	-0.058 (-0.135, 0.020)	-0.156 (-0.179, -0.134)	-0.137 (-0.255, -0.019)
Difference in slope between women and men, per y	-0.051 (-0.065, -0.036)	-0.144 (-0.192, -0.095)	0.051 (0.023, 0.079)	-0.082 (-0.199, 0.034)

ARIC, Atherosclerosis Risk in Communities. CHS, Cardiovascular Health Study. FOS, Framingham Offspring Cohort from Framingham Heart Study. NOMAS, Northern Manhattan Stroke study.

Interpretative Key: Global cognition measures global cognitive performance. All cognitive measures are set to a T-score metric (mean 50, SD 10); a 1-point difference represents a 0.1 SD difference in the distribution of cognition across the 5 cohorts. Higher cognitive scores indicate better performance. SBP= systolic blood pressure. NA=not applicable. y=year.

As a sensitivity analysis, we repeated analyses within the individual cohorts to assess heterogeneity in the associations between sex and cognitive decline. Linear mixed-effects models included time since first cognitive assessment and baseline values (measured before or at time of first cognitive assessment) of sex, race, age, years of school, alcohol use, cigarette smoking, body mass index, waist circumference, physical activity, time-varying cumulative mean systolic blood pressure (SBP), hypertension treatment, fasting glucose, low density lipoprotein (LDL) cholesterol, history of atrial fibrillation, age* follow-up time, sex* follow-up time, race* follow-up time, time-varying cumulative mean SBP* follow-up time, and hypertension treatment*follow-up time. To take into account correlation between longitudinal cognitive measures, we included random intercept and slope effects associated with subjects. All continuous variables were centered at the overall median, except cumulative mean SBP, which was centered at 120 mmHg. Glucose, LDL cholesterol, and SBP values were divided by 10 so that the parameter estimates refer to a 10-unit change in the variables. SBP was the time-dependent mean of all SBPs before the measurement of cognition. To estimate sex differences in cognitive decline, models included a sex*follow-up time interaction term.

The linear mixed-effect model for the CARDIA (Coronary Artery Risk Development in Young Adults) study with random intercept and random slope was non-identifiable owing to participants having up to two cognition visits.

© 2021 Levine DA et al. *JAMA Network Open.*

eTable 7: Time to Reach the Threshold of 0.5 Standard Deviation Decrease in Cognitive Function from Baseline by Sex

Cognitive outcomes	0.5 SD of mean baseline cognitive score, points	Slope in white men at median age, points per year	Slope in white women at median age, points per year	Time to reach threshold of 0.5 SD decrease in cognitive function from baseline, years	
				Men	Women
Global cognition	3.97	-0.21	-0.28	18.88	14.16
Executive function	4.22	-0.33	-0.39	12.79	10.82
Memory	3.29	-0.23	-0.234	14.28	14.04

The cognitive outcomes summarize cognitive test information across the cohorts and do not have established minimally clinically important difference (MCID) thresholds. Experts have defined clinically meaningful cognitive decline as a decline in cognitive function of 0.5 or more standard deviations (SD) from baseline cognitive scores[10]. Declines of 0.5 SD or greater from baseline cognitive scores correlate with a clinically meaningful decline in adults with normal cognition and dementia[11,12]. An informal approach is to compare the time to reach the threshold of a 0.5-SD decrease in cognitive function from baseline between women and men. **eTable 7** shows the time to reach the threshold of a 0.5-SD decrease in cognitive function from baseline by sex. Women will reach the thresholds of a 0.5-SD decrease from baseline cognitive scores faster than men for global cognition, executive function, and memory.

For example, the mean global cognition slope is -0.21 points per year in men and -0.28 points per year in women. It takes men, on average, 18.88 years and women 14.16 years for global cognition scores to decline 0.5 SD from the baseline score. Women will reach the threshold of a 0.5-SD decrease from the baseline score for global cognition 4.72 years faster than men. Similarly, women will have decreases of 0.5 SD from baseline scores 1.97 years faster for executive function and 0.24 years faster for memory. Based on this approach, the sex differences in cognitive declines are clinically meaningful.

© 2021 Levine DA et al. *JAMA Network Open.*

eTable 8: Association between Sex and Exclusion Because of History of Stroke or Dementia at Baseline or Incident Stroke or Incident Dementia Before First Cognitive Assessment

Excluded because of history of stroke or dementia at baseline or incident stroke pr incident dementia before first cognitive assessment	**Women (n= 15,034)**	**Men (n= 12,396)**	**P-value**
No	14,313 (95.2)	11,775 (95.0)	0.41
Yes	721 (4.8)	621 (5.0)	

© 2021 Levine DA et al. *JAMA Network Open.*

eReferences

1. Griffith L, van den Heuvel E, Fortier I, et al. AHRQ methods for effective health care. In: *Harmonization of Cognitive Measures in Individual Participant Data and Aggregate Data Meta-Analysis.* Rockville (MD): Agency for Healthcare Research and Quality (US); 2013.

2. Kolen M BR. *Test Equating: Methods and Practices.* New York: Springer; 1995.

3. Samejima F. Estimation of latent ability using a response pattern of graded scores. *Psychometrika.* 1970;35(1):1-17.

4. Asparouhov T, Muthen B. *Plausible Values for Latent Variables Using Mplus*; 2010.

5. Muthen LK, Muthen, B.O. *Mplus user's guide: Eighth Edition.* Los Angeles, CA: Muthen & Muthen; 1998-2017.

6. Thomson GH. The meaning of i in the estimate of g. *Britsh Journal of Psychology General Section.* 1934;25(1):92.

7. Thurstone LL. The vectors of mind: multiple-factor analysis for the isolation of primary traits. 1935.

8. Paramjit SG, Tim BS. Statistical analyses for Round Robin interaction data. *Canadian Journal of Statistics.* 2001;29(2):321-331.

9. Gross AL, Jones RN, Fong TG, Tommet D, Inouye SK. Calibration and validation of an innovative approach for estimating general cognitive performance. *Neuroepidemiology.* 2014;42(3):144-153.

10. Wolinsky FD, Unverzagt FW, Smith DM, Jones R, Stoddard A, Tennstedt SL. The ACTIVE cognitive training trial and health-related quality of life: protection that lasts for 5 years. *The journals of gerontology Series A, Biological sciences and medical sciences.* 2006;61(12):1324-1329.

11. Rossetti HC, Munro Cullum C, Hynan LS, Lacritz LH. The CERAD Neuropsychologic Battery Total Score and the progression of Alzheimer's Disease. *Alzheimer disease and associated disorders.* 2010;24(2):138-142.

12. Unger JM, van Belle G, Heyman A. Cross-sectional versus longitudinal estimates of cognitive change in nondemented older people: a CERAD study. Consortium to Establish a Registry for Alzheimer's Disease. *Journal of the American Geriatrics Society.* 1999;47(5):559-563.

© 2021 Levine DA et al. *JAMA Network Open.*
